# Increased epicardial adipose tissue is associated with left atrial mechanical dysfunction in patients with heart failure with mildly reduced and preserved ejection fraction

**DOI:** 10.1007/s00392-024-02466-7

**Published:** 2024-05-28

**Authors:** M. Lobeek, T. M. Gorter, B. D. Westenbrink, D. J. Van Veldhuisen, M. Rienstra

**Affiliations:** Department of Cardiology, University Medical Center Groningen, University of Groningen, Hanzeplein 1, PO Box 30.001, 9700 RB Groningen, The Netherlands

**Keywords:** Epicardial adipose tissue, Atrial mechanical dysfunction, HFmrEF, HFpEF, CMR

## Abstract

**Introduction:**

Heart failure (HF) with mildly reduced and preserved ejection fraction (HFmrEF/HFpEF) is often accompanied by atrial dysfunction. It has been suggested that specific ectopic fat depots, such as epicardial adipose tissue (EAT), may directly influence the myocardial cells and, therefore, be involved in the pathophysiology of atrial mechanical dysfunction. In this study, we aimed to investigate the association between EAT and left atrial (LA) mechanical dysfunction.

**Methods and results:**

In total, 82 patients with symptomatic HF and left ventricular ejection fraction > 40% were prospectively enrolled. All patients underwent CMR while in sinus rhythm. LA mechanical dysfunction was defined as the presence of LA end-systolic volume index > 52 mL/m^2^ and LA reservoir strain < 23%. EAT volume was indexed for body surface area. Mean age was 69 ± 10 years, 42 (51%) were women and mean body mass index (BMI) was 29 ± 6 kg/m^2^. Mean LVEF was 55 ± 9% and 34 (41%) patients had LA mechanical dysfunction. In patients with LA mechanical dysfunction, the EAT volume was significantly higher than in patients without LA mechanical dysfunction (90 vs 105 mL/m^2^, *p* = 0.02) while BMI was similar. In multivariable logistic regression analyses, increased EAT remained significantly associated with LA mechanical dysfunction (OR 1.31, 95% CI 1.03–1.66, *p* = 0.03).

**Conclusion:**

Increased EAT was associated with LA mechanical dysfunction in patients with HFmrEF and HFpEF. Further research is needed to elucidate the exact mechanisms that underlie this association.

**Supplementary Information:**

The online version contains supplementary material available at 10.1007/s00392-024-02466-7.

## Introduction

The prevalence rate of heart failure (HF) with mildly reduced or preserved ejection fraction (HFmrEF/HFpEF) is increasing to epidemic proportions, and this type of HF is significantly contributing to global morbidity and mortality [1]. Evidence suggests that HFmrEF and HFpEF have multiple overlapping pathophysiological mechanisms that all contribute to the development of the disease, including risk factors such as ageing, hypertension, diabetes mellitus and obesity [2]. Furthermore, in the course of the disease, enlargement of the left atrium (LA) and loss of its functions (as reservoir, conduit and ejection chamber) are hallmark findings [3, 4].

Obesity seems one of the most important comorbidities in the development of HFmrEF/HFpEF [5]. It also seems an important contributor to atrial dysfunction in these patients, possibly through activation of inflammatory and neurohumoral systems, as well as cellular-level modifications [6]. In this regard, especially epicardial adipose tissue (EAT) may be directly involved in the pathophysiology of atrial dysfunction in patients with HFmrEF/HFpEF [7]. EAT is the fat depot located in direct contact with the myocardium, and by this direct connection causing myocardial dysfunction [8, 9]. However, data on the relation between EAT and cardiac magnetic resonance imaging (CMR)-derived atrial dysfunction is scarce. We therefore investigated the association between EAT and LA mechanical dysfunction in patients with HFmrEF/HFpEF, using CMR.

## Methods

### Study population

All 125 patients included in the present study had symptomatic HF with New York Heart Association class II or III, left ventricular ejection fraction (LVEF) > 40% and N-terminal pro-hormone of brain natriuretic peptide (NT-proBNP) level > 125 ng/L. In addition, all patients needed to have echocardiographic evidence of left ventricular (LV) diastolic dysfunction, LA dilatation and/or LV hypertrophy [10].

The majority of the patients in this study (105, 84%) were enrolled in the Ventricular Tachyarrhythmia Detection by Implantable Loop Recording in Patients with Heart Failure and Preserved Ejection Fraction (VIP-HF) study and they provided written informed consent [11]. The remaining 20 patients were screened for the VIP-HF study, but did not meet all the inclusion and exclusion criteria, or refused the implantation of a loop recorder. The use of non-VIP-HF HF patients was approved by the local ethics committee. The investigation conforms with the principles outlined in the Declaration of Helsinki.

All patients underwent a standard diagnostic work-up for HF patients with LVEF > 40% at our centre, including medical history, physical examination, laboratory testing, echocardiography and CMR. For the present study, only patients who were in sinus rhythm during CMR assessment were included and patients with atrial fibrillation (AF) during CMR were excluded (*n* = 42). Patients were also excluded from the present analysis if no sufficient CMR images were available to assess LA parameters (*n* = 1).

### Cardiac magnetic resonance imaging

CMR was performed using a standard protocol for the acquisition of cardiac volume, function and mass. The used protocol is previously described [12]*.* In brief, CMR acquisitions were performed on a 1.5-T scanner (Siemens, Erlangen, Germany & Philips, Amsterdam, The Netherlands). Typical sequence parameters for the scanners were as follows: for Siemens: TE 1.08 ms; TR 700 ms; flip angle 40°; voxel size 2.3 × 2.3 × 6.0 mm; and for Philips: TE 0.93 ms; TR 2.1 ms; flip angle 25°; voxel size 1.6 × 1.8 × 8.0 mm. ECG-triggered cine loop images were obtained during breath hold at end-expiration using a retrospectively gated cine steady-state, free-precession sequence. Approximately 15 short-axis slices from base to apex were obtained, including the atria.

Cine loop images were analysed off-line by experienced observers using dedicated software (QMass 7.6 and 8.1, QStrain 2.0, Medis, Leiden, The Netherlands) [12]. Endo- and epicardial borders of the left and right ventricles were manually delineated on the end-diastolic and end-systolic phases on the short-axis stacks. End-diastolic volumes and end-systolic volumes were automatically calculated by the summation of slices multiplied by slice thickness method. Volumetric measurements were indexed for body surface area, according to the Mosteller formula [13]. Strain was measured as the total deformation of the myocardium from its baseline length to its maximum length, and is expressed as a percentage. Longitudinal LV strain and RV strain were measured on cine images. Using the long-axis slices, left and right atrial volumes were measured by tracing the area and length of both atria in end-systole and end-diastole. Atrial volume was approximated using the biplane area-length method. Subsequently, LA reservoir, conduit and contractile strain were assessed using cine long-axis two- and four-chamber acquisitions.

### Left atrial function

Left atrial mechanical dysfunction was defined as the combination of abnormal LA end-systolic volume indexed for body surface area and abnormal LA reservoir strain, both acquired from CMR. LA end-systolic volume index and LA reservoir strain were abnormal according to previously published cutoff values (i.e. > 52 mL/m^2^ for LA end-systolic volume index; and < 23% for LA reservoir strain) [14–16]. If none or only one of these measures was abnormal, LA mechanical dysfunction was not present.

### Epicardial adipose tissue

Epicardial adipose tissue was defined as the adipose tissue situated between the outer wall of the myocardium and the visceral layer of the pericardium [17, 18]. Epicardial adipose tissue was measured using the cine short-axis for contouring and the cine long-axis stacks for referencing. While delineating the short axis, cine long-axis images were available for reference to ensure accurate differentiation of the selected area. Short-axis T1 mapping at three levels (base, mid and apex) was used to confirm adipose tissue, as described previously [12]. After confirmation, the EAT part on the cine short-axis slices was extrapolated beyond the part that T1 maps were available. Epicardial adipose tissue volumes were calculated by summation of EAT volume of each slice using the modified Simpson’s rule and indexed for body surface area according to the Mosteller formula [13, 19].

### Statistical analysis

Continuous data are presented as mean ± standard deviation (SD) or median (interquartile range, IQR) depending on the distribution, while categorical data are presented as numbers (percentage). Patient characteristics between the patients with and without LA mechanical dysfunction were compared using an independent *T*-test or Mann–Whitney *U* test depending on their distribution, or a chi-square test or Fisher’s exact test for categorical variables.

Logistic regression analysis was used to assess the association between EAT and LA mechanical dysfunction. First, the association between EAT and LA mechanical dysfunction was univariably tested (model 1). Potential confounders/interacting covariates were selected based on the literature [20–22]. Multivariable regression analyses were used to adjust EAT for age, sex and body mass index (BMI) (model 2), and for adjustment for age, sex, BMI, LVEF, diabetes mellitus, history of AF, and myocardial infarction (model 3). Tests were performed with SPSS version 28 (IMB, Armonk, NY). A *p*-value < 0.05 was considered statistically significant.

## Results

### Patient characteristics

A total of 82 patients were included. The mean age was 69 ± 10 years, 42 (51%) were women, mean BMI was 29 ± 6 kg/m^2^ and 26 (32%) had a history of AF. In the selected patient group, one remaining patient had a history of ablation. Mean LVEF was 55 ± 9%, mean LA end-systolic volume index was 54 ± 19 mL/m^2^ and mean LA reservoir strain was 18 ± 9%. The characteristics of the study population are shown in Table 1.

**Table 1:** Patient characteristics

	All patients (*n* = 82)	No LA mechanical dysfunction (*n* = 48)	LA mechanical dysfunction (*n* = 34)	*p*-value
Age, years	69 ± 10	67 ± 11	71 ± 8	0.11
Female sex	42 (51%)	25 (52%)	17 (50%)	0.85
BMI, kg/m^2^	29 ± 6	29 ± 6	30 ± 5	0.69
LVEF ≥ 50%	58 (71%)	33 (69%)	25 (74%)	0.64
NYHA class				
- NYHA II	49 (60%)	30 (63%)	19 (56%)	0.57
- NYHA III	31 (38%)	17 (35%)	14 (41%)	0.57
History of AF	26 (32%)	14 (29%)	12 (35%)	0.56
Time between first AF diagnosis and CMR, months	16 (5–36)	15 (5–53)	17 (5–26)	0.92
Diabetes mellitus	26 (32%)	16 (33%)	10 (29%)	0.71
Hypertension	62 (76%)	37 (77%)	25 (73%)	0.71
Coronary artery disease	34 (42%)	19 (40%)	15 (44%)	0.68
COPD	15 (18%)	8 (17%)	7 (21%)	0.65
ATTR Amyloidosis	4 (5%)	1 (2%)	3 (9%)	0.44
CHA_2_DS_2_-VASc score	4 ± 1	4 ± 1	4 ± 1	0.97
Smoking	44 (54%)	27 (56%)	17 (50%)	0.22
- Past	37 (45%)	22 (46%)	44%)	
- Current	7 (9%)	5 (10%)	2 (6%)	
Alcohol use	25 (31%)	13 (27%)	12 (35%)	0.48
Vital signs				
Systolic blood pressure, mmHg	141 ± 22	142 ± 21	139 ± 23	0.56
Diastolic blood pressure, mmHg	72 ± 13	73 ± 13	70 ± 13	0.26
Heart rate, bpm	69 ± 10	69 ± 9	68 ± 11	0.72
Medications				
Beta blockers	70 (85%)	41 (85%)	29 (85%)	0.99
ACEi/ARB	57 (70%)	31 (65%)	26 (77%)	0.25
MRA	35 (43%)	24 (50%)	11 (32%)	0.11
Loop diuretics	68 (83%)	41 (85%)	27 (79%)	0.48
Statins	48 (59%)	30 (63%)	18 (53%)	0.39
Oral anticoagulation				
- VKA	16 (20%)	9 (19%)	7 (21%)	0.84
- NOAC	10 (12%)	7 (15%)	2 (9%)	0.43
Laboratory values				
NT-proBNP, pg/mL	890 (568–1916)	812 (509–1718)	987 (585–2280)	0.44
CRP, mg/L	4 (2–9)	3 (2–5)	4 (2–9)	0.21
Troponin T, ng/L	18 (13–33)	16 (13–30)	24 (12–43)	0.25
Echocardiography				
LVEF, %	54 ± 7	54 ± 8	53 ± 6	0.97
LV mass index, g/m^2^	112 ± 43	108 ± 38	117 ± 49	0.41
Mitral septal *e′* velocity, cm/s	6.3 ± 2.6	6.4 ± 3.0	6.1 ± 1.9	0.93
Mitral lateral *e′* velocity, cm/s	7.4 ± 2.2	7.4 ± 2.4	7.3 ± 2.1	0.83
LV *E*/*e′*	12.9 ± 5.4	11.6 ± 5.3	14.7 ± 5.2	**0.01**
LA volume index, mL/m^2^	40.5 ± 14.3	34.8 ± 8.6	48.8 ± 16.8	** < 0.001**
TAPSE, mm	21.3 ± 4.9	22.0 ± 4.5	20.1 ± 5.3	0.16
RV *s′*	12.3 ± 2.8	12.6 ± 2.7	11.9 ± 2.8	0.35
TR gradient	37.6 ± 13.8	33.6 ± 15.0	40.7 ± 12.2	0.15
Moderate mitral regurgitation	3 (4%)	12 (25%)	3 (9%)	0.07
CMR				
Total EAT indexed by BSA, mL/m^2^	96 ± 27	90 ± 24	105 ± 28	**0.02**
Ventricular EAT indexed by BSA, mL/m^2^	73 ± 21	69 ± 20	78 ± 22	0.06
Atrial EAT indexed by BSA, mL/m^2^	26 ± 13	24 ± 13	29 ± 12	0.11
LV ejection fraction, %	55 ± 9	55 ± 10	55 ± 9	0.75
LV end-diastolic volume, mL/m^2^	94 ± 25	91 ± 26	98 ± 22	0.22
LV mass, g/m^2^	61 ± 24	60 ± 21	63 ± 29	0.81
LV global longitudinal strain, %	18 ± 5	19 ± 5	18 ± 4	0.57
RV ejection fraction, %	57 ± 10	56 ± 10	57 ± 11	0.54
RV end-diastolic volume, mL/m^2^	83 ± 20	79 ± 20	88 ± 20	**0.03**
RV mass, g/m^2^	18 ± 5	17 ± 5	19 ± 5	0.07
LA end-diastolic volume index, mL/m^2^	33 ± 16	25 ± 9	46 ± 15	** < 0.001**
LA end-systolic volume index, mL/m^2^	54 ± 19	43 ± 14	70 ± 13	** < 0.001**
LA reservoir strain, %	18 ± 9	21 ± 9	13 ± 6	** < 0.001**
LA conduit strain, %	10 ± 5	11 ± 6	7 ± 4	**0.01**
LA contractile strain, %	9 ± 6	10 ± 6	6 ± 3	**0.001**
LA emptying fraction, %	38 ± 13	43 ± 14	32 ± 11	** < 0.001**
RA end-diastolic volume index, mL/m^2^	26 ± 15	22 ± 14	32 ± 15	** < 0.001**
RA end-systolic volume index, mL/m^2^	40 ± 18	35 ± 18	47 ± 16	** < 0.001**
RA emptying fraction, %	37 ± 13	39 ± 14	35 ± 13	0.14
RA reservoir strain, %	25 ± 13	25 ± 13	25 ± 12	0.82
RA conduit strain, %	10 ± 6	10 ± 6	11 ± 6	0.32
RA contractile strain, %	15 ± 9	15 ± 10	15 ± 8	0.73

*ACEi* angiotensin-converting enzyme inhibitor, *AF* atrial fibrillation, *ARB* angiotensin II receptor blocker, *ATTR* transthyretin amyloid, *BMI* body mass index, *COPD* chronic obstructive pulmonary disease, *NOAC* non-vitamin K antagonist oral anticoagulants, *EAT* epicardial adipose tissue, *LA* left atrium, *LV* left ventricle, *MRA* mineral corticoid receptor antagonist, *TAPSE* tricuspid annular plane systolic excursion, *RA* right atrium, *RV* right ventricle, *VKA* vitamin K antagonist. Bold values are statistically significant with a p-value < 0.05.

### Left atrial mechanical dysfunction

In 38 patients (46%), LA end-systolic volume was > 52 mL/m^2^, and in 62 patients (76%), LA reservoir strain was < 23%. In total, 34 patients (41%) had LA mechanical dysfunction according to our definition. Differences in characteristics between patients with and without LA mechanical dysfunction are also depicted in Table 1. Overall, most general characteristics were comparable between the two groups, including having a history of AF. Hereby, it should be noted that patients with AF at baseline were excluded from this analysis.

**Fig. 1:**
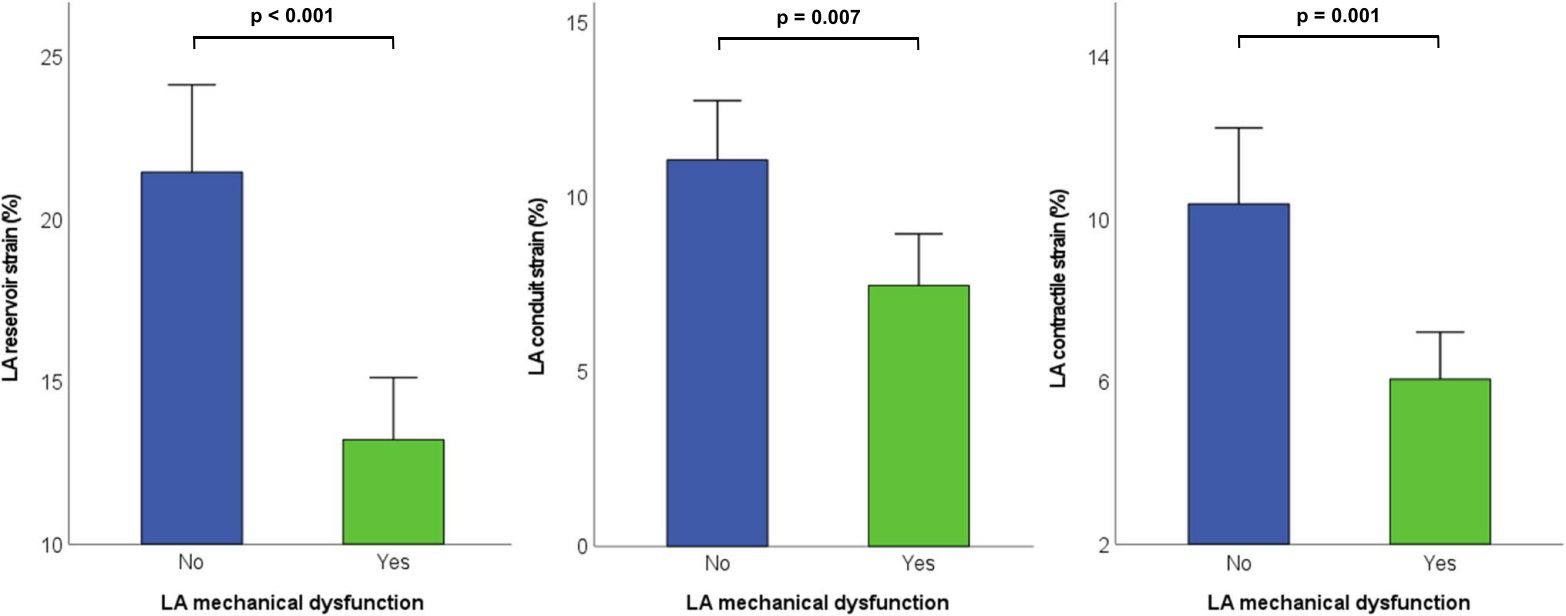
Differences in left atrial (LA) reservoir, conduit and contractile strain in patients with and without LA mechanical dysfunction

As expected, patients with LA mechanical dysfunction had also larger LA volumes on echocardiography, and lower LA conduit and contractile strain (Fig. 1). Patients with LA mechanical dysfunction had higher *E*/*e'* and larger RV and RA volumes.

**Fig. 2:**
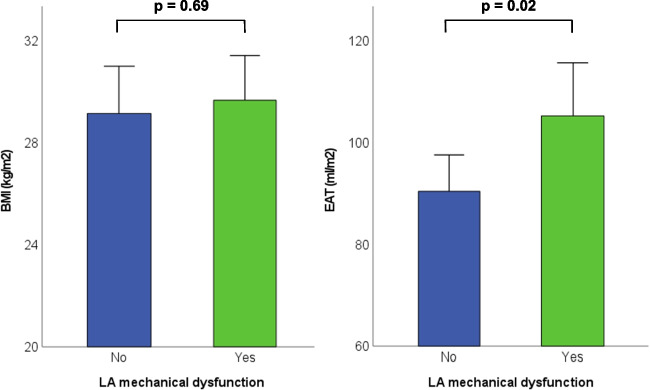
Differences in body mass index (BMI) and epicardial adipose tissue (EAT) in patients with and without LA mechanical dysfunction

### Association between EAT and LA mechanical dysfunction

Patients with LA mechanical dysfunction had a significantly higher amount of total EAT than patients without LA mechanical dysfunction (90 vs 105 mL/m^2^, *p* = 0.02), while BMI was similar between both groups (Fig. 2). The logistic regression analysis is shown in Table 2. In the unadjusted model (model 1), total EAT was significantly associated with LA mechanical dysfunction (OR 1.25, 95% CI 1.03–1.53, *p* = 0.02). After adjusting for either age, sex or BMI (model 2) as well as for age, sex, BMI, history of AF, diabetes mellitus, myocardial infarction and LVEF (model 3), the association between EAT and LA mechanical dysfunction remained significant. The location-specific analysis and analysis in only HFpEF patients have been added to the supplement as Supplementary Tables 1 and 2 respectively.

**Table 2:** Logistic regression analysis of EAT and LA mechanical dysfunction

EAT	OR (95% CI)	*p*-value
Model 1 (unadjusted)	1.25 (1.03–1.53)*	0.02
Model 2 (adjusted for age, sex, BMI)	1.28 (1.03–1.59)*	0.03
Model 3 (adjusted for age, sex, BMI, history of AF, DM, MI, LVEF)	1.31 (1.03–1.66)*	0.03

*ACMP* atrial cardiomyopathy, *AF* atrial fibrillation, *BMI* body mass index, *CI* confidence interval, *DM* diabetes mellitus, *EAT* epicardial adipose tissue, *LVEF* left ventricular ejection fraction, *MI* myocardial infarction, *OR* odds ratio

^*^Odds ratios given per 10-unit increase

## Discussion

In the present study, we showed that there is an association between EAT and LA mechanical dysfunction in patients with HFmrEF/HFpEF who were in sinus rhythm. Interestingly, this association was independent of BMI, potentially indicating that EAT plays a role in the pathophysiology of LA mechanical dysfunction in HFpEF irrespective of overall obesity.

Left atrial mechanical dysfunction is indicative of adverse LA remodelling, an important substrate for the development of AF [23]. The association between increased EAT and higher prevalence of AF has previously been shown [24]. However, only limited research has been conducted on the direct association between EAT and LA mechanical dysfunction. Evin et al. demonstrated in a small cohort of 19 controls and 20 non-HF patients with obesity and diabetes mellitus type 2 that LA strain measured by CMR was associated with EAT [25]. Additionally, Jin et al. also found an association between EAT thickness — measured by echocardiography — and LA and LV function in patients with HFmrEF/HFpEF [26]. However, only a modest correlation exists between EAT measured by echocardiography in comparison with CMR [27]. Our study presents the first data regarding the association between EAT and LA mechanical dysfunction in patients with HFmrEF/HFpEF, both measured by CMR.

Several mechanisms underlying the association between increased EAT and LA mechanical dysfunction have been postulated, such as increased inflammation, infiltration and accumulation of fibrosis as well as mechanical effects. Most mechanisms are predicated upon the observation that EAT and myocardial cells are directly connected without the presence of an intervening fascia, but the exact mechanisms remain poorly understood. Among others, the different mechanisms are elucidated in recent published reviews by our research group and Iacobellis [9, 28]. These reviews highlight that the direct interaction between EAT and the underlying myocardium enables EAT to communicate with the underlying myocardium. Therefore, it is possible that EAT may release harmful mediators to the myocardial cells, which infiltrate the ultrastructure of the myocardium and potentially lead to upregulation of inflammation [28]. The inflammatory characteristics of EAT itself may also lead to paracrine effects which cause fatty infiltration or accumulation of fibrosis [29, 30]. Next to these effects by mediators, there is evidence that EAT can induce mechanical effects by compressing the cardiac myocardium, leading to diastolic dysfunction and increased intracardiac pressures [31]*.* It is likely that a combination of factors reinforces each other, amplifying the influence of EAT on the heart. Considering these hypotheses, it would be reasonable to assume that EAT primarily exerts at local level and that atrial EAT plays a role in the onset and progression of LA remodelling, and therefore, in LA mechanical dysfunction. The present study did not show a significant association between atrial EAT and LA mechanical dysfunction. The amount of atrial EAT in our analysis included both left and right atrial EAT. Unfortunately, no data was available on LA EAT separately, which could be one of the possible explanations of the non-significant association between atrial EAT and LA mechanical dysfunction. Other explanations can be found within the mechanisms underlying the association between EAT and myocardial dysfunction, where the exact pathophysiological mechanisms of EAT remain unclear. Hypothetically, EAT surrounding the entire heart may also have more effect on LA mechanical dysfunction through hemodynamic mechanical effects than local atrial EAT. The lack of significance in the association between atrial EAT and LA mechanical dysfunction could also be attributed to varying contributions of the different hypotheses proposed.

Building upon LA remodelling, there has been a growing interest in atrial cardiomyopathy, whose exact definition is yet to be determined [3]. Kreimer and Gotzmann have proposed a model categorizing atrial cardiomyopathy into three components, i.e. mechanical dysfunction, atrial fibrosis and electrical dysfunction [32]. In this study, our focus was on CMR-derived LA mechanical dysfunction, as we encountered limitations in assessing LA fibrosis and electrical dysfunction. Specifically, our imaging sequences were not suitable for evaluating LA fibrosis, and we did not conduct voltage maps, which are required for assessing LA fibrosis. Furthermore, our standard 12-lead ECGs were inadequate for measuring electrical dysfunction, as they lacked the necessary digitally stored data for post-processing with specific software. We defined LA mechanical dysfunction as the combination of LA end-systolic volume index and LA reservoir strain. LA end-systolic volume index is a widely used parameter and is one of the most important determinants of atrial remodelling [33]. Additionally, LA strain provides insights into the functionality of the LA [34]. Since the presence of AF lasting longer than 48 h leads to the possibility of LA stunning and subsequently lower LA strain values, only patients in sinus rhythm were included [14]. The chosen cutoff values of LA end-systolic volume index and LA reservoir strain are based on previously published values [14–16]. However, due to the relative novel modality, normal ranges of both parameters in CMR still need to be confirmed [35–37].

In future research, it would be intriguing to combine all three described components of atrial cardiomyopathy to further explore this definition and evaluate the association between EAT and atrial function.

### Limitations

First, the relative modest sample size limits the ability to adjust for multiple confounders. The relative modest sample size also limits the statistical power of the cohort, especially when focusing solely on HFpEF patients. Second, patients with implantable cardiac devices were excluded from the study, which potentially limits the generalizability of the findings. Third, scanners from two different vendors were used for the CMR acquisitions. Albeit the vast majority of scans were performed on a single Siemens scanner (*n* = 74, 90%), we anticipate minimal impact on the results, especially regarding the measurements of the LA size and function and the volume of EAT. Fourth, the cross-sectional nature of the study limits the investigation of a causal relation between increased EAT and LA mechanical dysfunction.

## Conclusion

Increased EAT was associated with LA mechanical dysfunction in patients with HF with mildly reduced or preserved ejection fraction. Further research is needed to elucidate the exact mechanisms that underlie the association between EAT and LA mechanical dysfunction.

## Supplementary Information 

ESM1 Supplementary file1 (DOCX 13.2 KB)

ESM2 Supplementary file2 (DOCX 13.3 KB)
