# Supplementary material for: Increased epicardial adipose tissue is associated with left atrial mechanical dysfunction in patients with heart failure with mildly reduced and preserved ejection fraction
Source: Clin Res Cardiol. 2024 May 28;114(5):601–8. doi: 10.1007/s00392-024-02466-7 (PMC12058962; doi:10.1007/s00392-024-02466-7)
Supplement: Supplementary file 1 — Supplementary file1 (DOCX 13.2 KB) [file 392_2024_2466_MOESM1_ESM.docx]

***Supplementary Table 1:*** ***Logistic regression analysis of atrial EAT and LA mechanical dysfunction***

| **Atrial EAT** | **OR [95% CI]** | **p-value** |
| --- | --- | --- |
| Model 1 (Unadjusted) | 1.32 [0.91-1.92]* | 0.15 |
| Model 2 (Adjusted for age, sex, BMI) | 1.37 [0.93-2.04]* | 0.12 |
| Model 3 (Adjusted for age, sex, BMI, history of AF, DM, MI, LVEF) | 1.38 [0.92-2.08]* | 0.12 |

AF = atrial fibrillation, BMI = body mass index, CI: confidence interval, DM = diabetes mellitus, EAT = epicardial adipose tissue, LVEF = left ventricular ejection fraction, MI = myocardial infarction, OR: Odds Ratio.

* Odds ratios given per 10-unit increase
