# Supplementary material for: Increased epicardial adipose tissue is associated with left atrial mechanical dysfunction in patients with heart failure with mildly reduced and preserved ejection fraction
Source: Clin Res Cardiol. 2024 May 28;114(5):601–8. doi: 10.1007/s00392-024-02466-7 (PMC12058962; doi:10.1007/s00392-024-02466-7)
Supplement: Supplementary file 2 — Supplementary file2 (DOCX 13.3 KB) [file 392_2024_2466_MOESM2_ESM.docx]

***Supplementary Table 2: Logistic regression analysis of EAT and LA mechanical dysfunction in patients with LVEF above 50 percent (n=58)***

| **EAT** | **OR [95% CI]** | **p-value** |
| --- | --- | --- |
| Model 1 (Unadjusted) | 1.15 [0.94-1.42]* | 0.18 |
| Model 2 (Adjusted for age, sex, BMI) | 1.16 [0.91-1.50]* | 0.24 |
| Model 3 (Adjusted for age, sex, BMI, history of AF, DM, MI, LVEF) | 1.23 [0.93-1.62]* | 0.15 |

AF = atrial fibrillation, BMI = body mass index, CI: confidence interval, DM = diabetes mellitus, EAT = epicardial adipose tissue, LVEF = left ventricular ejection fraction, MI = myocardial infarction, OR: Odds Ratio.

* Odds ratios given per 10-unit increase
